# Multidisciplinary team meetings for lung cancer in the Nordic countries: results from a Nordic survey

**DOI:** 10.2340/ao.v65.45213

**Published:** 2026-04-17

**Authors:** Anja Gouliaev, Janna Berg, Johan Isaksson, Heidi Andersén, Torben Riis Rasmussen

**Affiliations:** a Department of Respiratory Diseases and Allergy, Aarhus University Hospital, Aarhus N, Denmark; b Department of Clinical Medicine, Aarhus University, Aarhus N, Denmark; c Department of Pulmonology, Vestfold Hospital Trust, Tønsberg, Norway; d Centre for Research and Development, Region Gävleborg, Uppsala University, Uppsala, Sweden; e Cancer Center, Vaasa Central Hospital, Vaasa, Finland; f Department of Clinical Oncology, University of Turku, Turku, Finland; g Faculty of Medicine and Health Technology, Tampere University, Tampere, Finland

**Keywords:** Lung cancer, Scandinavian and Nordic countries, multidisciplinary team conference, tumor board, healthcare survey

## Introduction

The management of lung cancer is complex and requires coordinated input from multiple specialties. Multidisciplinary team (MDT) meetings provide a structured forum in which respiratory physicians, oncologists, thoracic surgeons, radiologists, pathologists, and specialist nurses review diagnostic findings and formulate treatment strategies [1–3]. MDT-based care has been associated with improved staging accuracy and guideline adherence in lung cancer [4–6]. Effective MDT decision-making relies on access to relevant information, structured case presentations, clear leadership, and an infrastructure that supports balanced participation among team members. While MDT meetings are widely implemented across Europe, there is no international consensus defining how they should be organized, which patients should be discussed, or how meeting processes should be structured. The Nordic countries share publicly financed healthcare systems with universal access and established lung cancer registries, yet operational organization of MDT meetings may vary across settings [7].

We therefore conducted a cross-national survey to describe and compare lung cancer MDT meetings in Denmark, Finland, Norway, and Sweden, with particular focus on governance, meeting frequency, patient selection, and perceived barriers and drivers. Identifying structural similarities and differences may inform future operational guidance at national or Nordic level.

## Patients/material and methods

After the turn of the century, MDT meetings have been implemented in all four Nordic countries. A lung cancer MDT meeting was defined as a formal multidisciplinary forum in which diagnostic and treatment decisions for lung cancer patients are discussed.

This was a non-interventional cross-sectional survey conducted between June and September 2025.

A senior respiratory physician functioning as a leader of lung cancer MDT meetings from each of the four participating countries, were invited to participate in the development of the survey questions and to distribute the survey nationally. All lung cancer MDT meetings in Denmark (*n* = 7), regional MDT meetings in Norway (*n* = 7), and treating hospitals in Finland (*n* = 20) and Sweden (*n* = 21) were invited to participate. In Norway, only regional MDT meetings were included to ensure comparable case complexity and access to thoracic surgery. One reminder was sent after 2 weeks.

### Survey instrument

The questionnaire comprised 53 items addressing:

- MDT composition and leadership
- Governance and guideline support
- Meeting frequency and duration
- Case volume and patient selection
- Infrastructure and remote participation
- Biomarker testing
- Perceived barriers and drivers

Full questionnaire details are provided in Supplementary Material.

### Statistical approach

Quantitative responses were summarized using descriptive statistics.

## Results

### Response rate and MDT characteristics

Thirty of the 55 (55%) invited centers responded: Denmark 7/7 (100%), Finland 6/20 (30%), Norway 6/7 regional MDTs (86%), and Sweden 11/21 (52%).

Respiratory physicians chaired 90% of MDT meetings. Ninety-three percent reported local or national MDT guidelines. Sixty percent used structured templates for case presentation, and 62% reported access to second-opinion MDT meetings (Table 1).

**Table 1:** An overview of lung cancer MDT in the Nordic countries and barriers and drivers for MDT.

	Denmark	Finland	Norway	Sweden	Total
Responders	7/7 (100%)	6/20 (30%)	6/7 (86%)	11/21 (52%)	30/55 (55%)
MDT guideline local/national	100%	100%	86%	91%	93%
Guideline support (1–10)	8–10	5–9	5–10	5–10	5–10
Leader of MDT	Respiratory physician	Respiratory physician/ oncologist	Respiratory physician	Respiratory physician/ oncologist/ thoracic surgeon	90% respiratory physician
Frequency of MDT	Multiple times a week	Multiple times a week – weekly	Multiple times a week – weekly	Multiple times a week – weekly	Multiple times a week – weekly
Duration of MDT	30–120 min	30–120 min	30–90 min	< 30 – > 120 min	< 30 – > 120 min
Average no. of patients per MDT	5 – > 25	1–19	5–14	5–25	1 – > 25
Time from diagnosis to MDT	< 3 days – 2 weeks	< 3 days – > 2 months	< 3 – 7 days	< 3 days to 3–4 weeks	< 3 days – > 2 months
Proportion of patients not discussed at MDT	< 10–29%	< 10–69%	< 10–49%	< 10–29%	<10–69%
MDT template used	100%	50%	100%	45%	60%
2^nd^ opinion MDT	43%	83%	50%	73%	62%
**Barriers for MDT (*n* = 24)**			**Drivers for MDT (*n* = 28)**		
Available time 13 (54%) All countries Complete team 4 (17%) Finland, Norway IT-equipment 4 (17%) Denmark, Norway, Sweden Room setting 2 (7%) Finland, Norway			Multidisciplinary input required 27 (96%) All countries Case complexity 22 (79%) All countries Per national guidelines 21 (75%) All countries Per quality standard 19 (68%) All countries Per local guideline 8 (29%) Denmark, Norway, Sweden		

MDT: multidisciplinary team.

### Meeting frequency and workload

Meeting frequency ranged from weekly to multiple times per week (Table 1). Danish centers reported multiple weekly meetings, whereas most Finnish and Norwegian centers met weekly. The number of cases per meeting ranged from 1 to more than 25. Meeting duration ranged from 30 min to more than 2 h with average case numbers between one to 25 patient cases. Most centers use a standard template for case presentation and some have the option to refer for a second opinion MDT. Most respondents reported that both the number of cases discussed and total time spent in MDT meetings had increased over the past 3–5 years. Remote or hybrid participation was reported in all countries but varied considerably between centers.

### Patient selection

**Figure 1:**
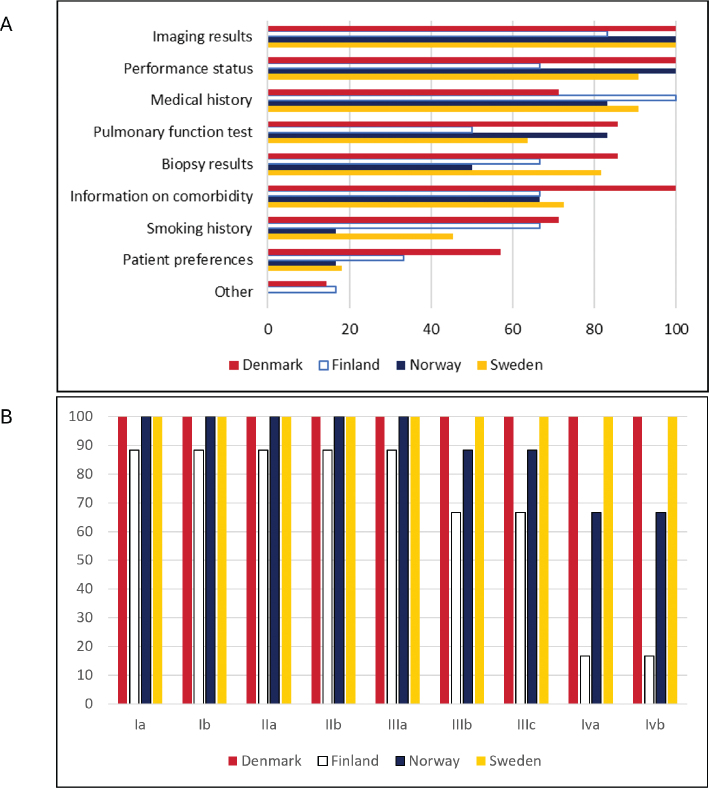
(A) Information required for the case to be presented at the lung cancer MDT in percent, by country (30 answers) (B) Stages of NSCLC included in discussions at MDT in percent, by country (30 answers).

Patient information required to be assessed at lung cancer MDT meeting is shown in Figure 1A. Variation in patient selection was observed (Figure 1B). All Danish centers and most Swedish centers reported discussing lung cancer patients across all disease stages. In contrast, several Finnish and Norwegian centers reported more selective discussion, particularly focusing on patients eligible for surgery or curative treatment.

The proportion of patients not discussed at MDT meetings ranged from < 10% to up to 69% across centers.

### Perceived barriers and drivers

The most frequently reported driver for MDT meetings was the need for multidisciplinary input (96%), followed by case complexity (79%) and national guideline requirements (75%). Insufficient available time was identified as a barrier by 54% of respondents across all countries. Additional barriers included IT limitations (17%) and incomplete team participation (17%). Most lung cancer MDT centers have national or local guidelines, which to some extent support the MDTs’ role in decision-making (Table 1).

## Discussion and conclusion

This study provides a contemporary overview of lung cancer MDT meetings in four Nordic countries. Despite broadly similar healthcare systems and guideline frameworks, we identified considerable variation in meeting frequency, case volume, and patient selection. Professional composition and leadership were largely consistent, with respiratory physicians chairing most meetings. The widespread presence of local or national guidelines suggests institutionalized MDT practice.

Meeting frequency and case volume varied substantially. Danish centers reported more frequent meetings and higher case numbers, whereas other countries more often conducted weekly meetings. Prior research suggests that effective MDT performance depends on regular meetings, manageable caseloads, and structured presentation formats [1–3, 8]. The relatively low number of cases discussed per MDT meeting in the Nordic countries aligns with previous studies on lung cancer MDTs [7] and MDTs for other cancers [8] in a Nordic setting. In contrast, MDTs in the United Kingdom often review more than 20 patients per meeting [9, 10], a volume associated with decision making fatigue [11]. Given that most Nordic lung cancer MDTs discuss fewer than 20 cases, this is unlikely to be a concern.

Prolonged diagnostic interval, exceeding 2 months in one Finnish center, highlights a challenge in delayed treatment. Another challenge in Finland is the limited access to computed tomography scans at primary care centers, which are often the first point of contact for patients who exhibit symptoms [12].

Variation in patient selection was notable. While some centers discussed all disease stages, others focused on potentially curative cases. Stage III non-small cell lung cancer (NSCLC), representing locally advanced disease, frequently presents diagnostic and therapeutic challenges [13–15]. Treatment intent for stage III NSCLC patients can be either curative or palliative depending on minor differences. In two Finnish and one regional Norwegian lung cancer centers, stage IIIB and IIIC patients were not routinely presented at MDT meetings. Registry data show that 91% of all Danish lung cancer patients and 93% of curatively treated lung cancer patients in Norway were discussed at local and regional MDT meeting in 2024 [16, 17] and 83% of all Swedish lung cancer patients in 2023 [18]. Finland lacks comparable national registry data [19]. By comparison, the United Kingdom has shifted toward more selective MDT discussion to streamline workflow [20, 21]. Whether all lung cancer patients should be routinely discussed remains debated. Selective discussion may improve efficiency but risk variation in care pathways. Clearer operational guidance may help to balance comprehensiveness and feasibility.

Although several benefits of MDT-based lung cancer care were highlighted, our findings also suggest barriers to optimal MDT function in the Nordic countries. National or local guidelines typically define roles, responsibilities, and eligibility, but often lack operational structure such as meeting workflow, documentation standards, or leadership responsibilities. In some centers, MDT structures were established before national guidelines, limiting the impact of later recommendations. To improve lung cancer MDTs in the Nordic countries, we propose developing national or Nordic-level operational guidelines and implementing routine MDT audits.

### Strengths and limitations

To our knowledge, this is the first cross-national survey describing lung cancer MDT meetings across the Nordic countries. However, uneven response rates and differences in inclusion criteria, such as inclusion of only regional MDT meetings in Norway, limit cross-country comparability. The Finnish response rate was low. Data were self-reported and not validated against registry data or direct observation. Furthermore, we did not assess clinical outcomes, consensus rates, or documentation of staging decisions.

## Conclusion

Nordic lung cancer MDT meetings share a similar multidisciplinary composition, but differ in meeting frequency, workload, and patient selection. Increasing demands and variation in operational structure highlight the need for clearer organizational guidelines to support sustainable and consistent MDT-based lung cancer care.

## Supplementary Material



## Data Availability

Data are available on request from the corresponding author.
