# Supplementary material for: Multidisciplinary team meetings for lung cancer in the Nordic countries: results from a Nordic survey
Source: Acta Oncol. 2026 Apr 17;65:45213. doi: 10.2340/ao.v65.45213 (PMC13097091; doi:10.2340/ao.v65.45213)
Supplement: Supplementary file 1 [file AO-65-45213-s1.pdf]

Supplementary material has been published as submitted. It has not been copyedited, or typeset by Acta Oncologica

Lung cancer MDTs in the Nordics

A multinational survey study of the implementation of multi-disciplinary team meetings across Sweden, Norway, Finland, and Denmark

Please state your country:

Please state your hospital:

1. Is there a guideline (national or local) for MDT for lung cancer patients at your hospital?
2. To what extent do guidelines generally support or hinder MDTs' role in decision-making?

    Considerable hinder 1, 2, 3, 4, 5, 6, 7, 8, 9, 10 Considerable support

3. Please elaborate on how guidelines hinder/support the MDTs' role in decision making
4. To what extent do guidelines affect which medical specialties are involved in MDT?

    No effect 1, 2, 3, 4, 5, 6, 7, 8, 9, 10 Large effect

5. Please explain any impact of guidelines on medical specialties involved in MDT?
6. To what extent has your MDT modified national or local guidelines to better suit your specific clinical practice?

    No modification 1, 2, 3, 4, 5, 6, 7, 8, 9, 10 Considerable modification

7. How should guidelines be structured to best support the role of MDTs?
8. Who is the principal lead in your local MDT?

    Respiratory physician, oncologist, radiologist, thoracic surgeon, pathologist,

    other

9. How often does the lung cancer MDT meet (in person or virtual)?

    Never, annually, quarterly, monthly, weekly, multiple times a week

10. What average proportion of your MDT meetings are with some level of remote/video participation

    <10%, 10-29%, 30-49%, 50-69%, 70-89%, >90%

11. How often does your MDT collaborate with specialists/MDTs from other hospitals?

    Never, annually, quarterly, monthly, weekly, multiple times a week

12. What is the MDT meeting duration on average

<30 min, 30-59 min, 60-89 min, 90-120 min, >120 min

13. How many cases do you discuss on average per MDT meeting

1-4, 5-9, 10-14, 15-19, 20-25, >25

14. What is the time from diagnosis of lung cancer to the first MDT discussion of the patient?

<3 days, 4-7 days, 1-2 weeks, 3-4 weeks, 1-2 months, >2 months

15. What is the minimum information that can be presented to the MDT to discuss a patient? (select ***only*** those that are ***strictly required***)

Performance status, biopsy results, imaging results, pulmonary function test, medical history, information on comorbidity, smoking history, patient wishes, other

16. Please select all stages of NSCLC that are included in discussions at your MDTs

Ia, Ib, IIa, IIb, IIIa, IIIb, IIIc, Iva, IVb

17. Please explain if staging impacts the need for discussion at an MDT

18. What proportion of patients are not discussed at an MDT?

<10%, 10-29%, 30-49%, 50-69%, 70-89%, >90%

19. What are the most common reasons for ***not*** taking a patient to an MDT meeting?

Time constraints, urgency of treatment initiation, no need (clear decision on stage and treatment), other

20. What are most common characteristics of patients ***not*** discussed by the MDT?

Poor prognosis, best supportive care only, poor performance status, lack of essential information to assess, other

21. If there is no formal MDT-discussion of a patient: in what other ways are doctors from other specialties involved in guiding decision-making?

22. Who presents patient cases?

Oncologist, pathologist, thoracic surgeon, respiratory physician, nurse specialist, junior doctor, other

23. Do you use a standard template for case presentation?

Yes, no, sometimes

24. Are there written case summaries prior to MDT?

Yes, no, sometimes

25. Who is responsible for writing the case summary?

Nurse, medical secretary, oncologist, pathologist, thoracic surgeon, pulmonologist, radiologist, other

26. How are the results of the MDT discussion documented for each patient?

27. Who is responsible for the documentation process?

Nurse, medical secretary, oncologist, pathologist, thoracic surgeon, pulmonologist, radiologist, other

28. Are some patients referred for 2nd opinion MDT?

    Yes, no

29. If yes to above, please elaborate
30. When are patients discussed at an MDT? (select all that apply)

    Pre-treatment, during treatment, post-treatment

31. What most commonly drives the need for assessment after treatment initiation
32. What barriers, if any, do you experience for having MDT?

    Available time, complete team, setting, IT-equipment, collegial support, managerial support, other

33. What drivers do you experience that are supportive of MDTs?

    Case complexity, multidisciplinary input required, per accreditation/quality standard, per national guidelines, per local guidelines, other

34. How has the number of cases discussed at an MDT evolved over the last 3-5 years?

    Decreased significantly, decreased slightly, unchanged, increased slightly, increased significantly

35. How has the total “MDT time” evolved over the last 3-5 years?

    -Decreased significantly, decreased slightly, unchanged, increased slightly, increased significantly

36. In what way has MDT involvement in diagnosis and treatment decisions changed in the last 3-5 years?
37. In what way do you expect the MDT’s role to change in the next 3-5 years?
38. What proportion of patients in stage I have biomarker tests performed?

    <10%, 10-29%, 30-49%, 50-69%, 70-89%, >90%

39. What proportion of patients in stage II have biomarker tests performed?

    <10%, 10-29%, 30-49%, 50-69%, 70-89%, >90%

40. What proportion of patients in stage III have biomarker tests performed?

    <10%, 10-29%, 30-49%, 50-69%, 70-89%, >90%

41. What proportion of patients in stage IV have biomarker tests performed?

    <10%, 10-29%, 30-49%, 50-69%, 70-89%, >90%

42. Who orders the test?

    Oncologist, pathologist, thoracic surgeon, pulmonologist, radiologist, nurse, other

43. What is the typical time between a biomarker test being ordered and the test result being received by the MDT and/or the requesting physician?

<2 days, 3-4 days, 5-6 days, 1-2 weeks, 3-4 weeks, >4 weeks

44. What is the time between diagnosis of NSCLC and availability of complete biomarker test results?

<1 week, 1-2 weeks, 3-4 weeks,5-6 weeks, >6 weeks

45. Which biomarkers are typically ordered for NSCLC patients?

PD-L1, EGFR, KRAS, BRA, ROS1, other

46. What technology is most commonly used?

Single-gene or NGS, IHC, FISH, PCR

47. How often are biomarker results ready prior to your MDT discussions?

<10%, 10-29%, 30-49%, 50-69%, 70-89%, >90%

48. For what proportion of patient cases discussed in MDTs are ready biomarker results actually used?

<10%, 10-29%, 30-49%, 50-69%, 70-89%, >90%

49. What are the most common barriers/issues in regard to having biomarker test results available to MDT?

<10%, 10-29%, 30-49%, 50-69%, 70-89%, >90%

50. Who presents the results of biomarker tests in your MDTs

Oncologist, pathologist, pulmonologist, other

51. To what extent do biomarker results play a role in treatment decisions for stage I NSCLC patients?

Very small role 1, 2, 3, 4, 5, 6, 7, 8, 9, 10 Very large role
